# Felons

**Published:** 1874-06

**Authors:** 


### FELONS

are of two kinds—one under the skin, readily cured, the other immediately on the bone and under the fascia, or whit-leather, which is tougher than India rubber.

The felon, being in the nature of a boil, swells, rises up, but the fascia being so impenetrable, cannot break as the skin does, hence the pain is more intolerable. A boil or superficial felon, rises, pierces the skin, breaks, runs itself out and gets well. The matter of a bone felon cannot escape, but remains in its place, irritates, inflames and gives a pain second only to the rack; but pressure and heat cause the matter to be absorbed at last, and the martyred wretch gets well slowly, and after a long time.

There are two ways of treating a felon, as there are two kinds of felons. If superficial, put a fly blister on the spot at once about the size of the thumb nail; let it remain six hours, when the felon or core may be seen immediately under the blister, and may be picked out with the point of a needle.

The London *Lancet* is in error when it gives this as a cure for a bone felon. The best and speediest remedy is to make a cross cut down to the bone with a lancet, until the point is felt to scrape on the bone; this lets out the yellow matter at once, and relief is instantaneous. This ought to be done as soon as the finger begins to hurt and throb. A felon is caused by a bruise.

